# Fibroblast activation protein-α expression in fibroblasts is common in the tumor microenvironment of colorectal cancer and may serve as a therapeutic target

**DOI:** 10.3389/pore.2023.1611163

**Published:** 2023-08-08

**Authors:** K. Greimelmaier, N. Klopp, E. Mairinger, M. Wessolly, S. Borchert, J. Steinborn, K. W. Schmid, J. Wohlschlaeger, F. D. Mairinger

**Affiliations:** ^1^ Institut für Pathologie, Diakonissenkrankenhaus Flensburg, Flensburg, Germany; ^2^ Institut für Pathologie, Universitätsklinikum Essen, Essen, Germany

**Keywords:** colorectal cancer, tumor microenvironment, cancer-associated fibroblasts, fibroblast-activation protein, radioligand therapy

## Abstract

**Background:** Colorectal cancer (CRC) is still one of the leading causes of cancer death worldwide, emphasizing the need for further diagnostic and therapeutic approaches. Cancer invasion and metastasis are affected by the tumor microenvironment (TME), with cancer-associated fibroblasts (CAF) being the predominant cellular component. An important marker for CAF is fibroblast activation protein-α (FAP) which has been evaluated as therapeutic target for, e.g., radioligand therapy. The aim of this study was to examine CRC regarding the FAP expression as a candidate for targeted therapy.

**Methods:** 67 CRC, 24 adenomas, 18 tissue samples of inflammation sites and 28 non-neoplastic, non-inflammatory tissue samples of colonic mucosa were evaluated for immunohistochemical FAP expression of CAF in tissue microarrays. The results were correlated with clinicopathological data, tumor biology and concurrent expression of additional immunohistochemical parameters.

**Results:** 53/67 (79%) CRC and 6/18 (33%) inflammatory tissue specimens showed expression of FAP. However, FAP was only present in 1/24 (4%) adenomas and absent in normal mucosa (0/28). Thus, FAP expression in CRC was significantly higher than in the other investigated groups. Within the CRC cohort, expression of FAP did not correlate with tumor stage, grading or the MSI status. However, it was observed that tumors exhibiting high immunohistochemical expression of Ki-67, CD3, p53, and β-Catenin showed a significantly higher incidence of FAP expression.

**Conclusion:** In the crosstalk between tumor cells and TME, CAF play a key role in carcinogenesis and metastatic spread. Expression of FAP was detectable in the majority of CRC but nearly absent in precursor lesions and non-neoplastic, non-inflammatory tissue. This finding indicates that FAP has the potential to emerge as a target for new diagnostic and therapeutic concepts in CRC. Additionally, the association between FAP expression and other immunohistochemical parameters displays the interaction between different components of the TME and demands further investigation.

## Introduction

Colorectal cancer (CRC) is one of the most common malignancies and accounts for 9.2% of cancer-related deaths worldwide [1]. The majority of cancer patients die from metastatic disease. Despite increased efforts in screening programs, more than half of the patients with CRC show advanced disease and metastases at the time of diagnosis [2], emphasizing the need for further therapeutic approaches. Cancer invasion and metastatic spread are affected by different cells and pathological reactions in the tumor microenvironment (TME). Among tumor stromal cell types, cancer-associated fibroblasts (CAF) proved to be the predominant component in TME [3, 4]. Fibroblasts in general synthesize the extracellular matrix (ECM) of connective tissue and are essential for tissue repair after damage. Activated fibroblasts in the TME, i.e., activated CAF contribute to the regulation and initiation of crucial steps for malignant progression of tumors. Especially in tumors with high levels of desmoplasia such as pancreatic ductal adenocarcinoma and colorectal adenocarcinoma, recruitment and activation of CAF play a key role in carcinogenesis [5, 6]. CAF can produce and modulate the ECM of tumors and are involved in the recruitment of other cell types and the production of different enzymes, immunomodulatory cytokines and growth factors [7, 8]. They are able to induce invasive growth and tumor proliferation by promoting epithelial-mesenchymal transition of tumor cells as well as initiate metastasis [9–11].

Although the origin of CAF and the mechanism of transformation from normal fibroblasts to activated CAF remains unknown, there are several markers to identify CAF in the TME [12]. Among α-smooth muscle actin (SMA), and platelet-derived growth factor receptor (PDGF-R), fibroblast activation protein-α (FAP) proved to be a reliable marker for activated CAF [3, 4, 8, 13]. FAP is a type II transmembrane glycoprotein of the group of plasma membrane-bound serine proteases that is upregulated in fibroblasts at sites of active tissue remodeling, including wound healing and fibrosis. While FAP is absent in normal tissue [14–16], high expression of FAP has been detected in several solid tumors such as CRC [8, 13, 17]. Previous studies have demonstrated that high FAP expression is associated with an increased risk of metastases and poor survival in CRC and thus proposed FAP as a possible biomarker for disease progression [4, 8, 17, 18]. Furthermore, FAP has been discussed as both a novel therapeutic target and diagnostic tool. Although there are some studies examining the use of FAP-targeted radioligands for *in-vivo* imaging and targeted nucleotide therapy for a variety of cancers, including CRC [19–22], this field of research has yet to be fully developed.

The aim of this study was to examine CRC regarding the expression of FAP as a potential candidate for targeted imaging and therapy and to correlate the results with other immunohistochemical parameters in the TME such as p53, β-catenin, Ki-67 and CD3.

Certain aspects of this study have already been presented at a conference [23].

## Materials and methods

### Identification of patients and biopsies

A total of 76 colorectal adenocarcinomas from 72 adult patients that underwent radical surgical resection at University Hospital Essen in the period from 2001 to 2020 were included in this study. The collected specimens encompassed colorectal adenocarcinomas with pathological stages ranging from pT1-4b, pN0-2b, pM0-1, and grading G1-3. Furthermore, 24 colorectal adenomas with low-grade or high-grade dysplasia were included for evaluation. In addition, samples of inflamed tissue from the colorectal tract (i.e., diverticulitis, colitis) as well as non-neoplastic, non-inflammatory tissue (e.g., cases of diverticulosis) were examined as control cohorts.

### Histological review and tissue microarrays (TMA)

Formalin-fixed, paraffin-embedded tissue were retrieved from the archives of the Department of Pathology of the University Essen. Four µm sections were cut and stained with haematoxylin and eosin to define representative tumor regions (i.e., tumor center). Two core tissue biopsies with a diameter of 0.6 mm were punched from selected areas of each case using a thin-wall stainless steel tube and brought into a new paraffin block. Evaluation of the TMA confirmed that the tumor tissue cores correspond to the original block. Cores without viable tumor or artefacts were excluded. All specimens were reviewed by two experienced pathologists (KG and JW).

### Immunohistochemistry

Tissue sections of thickness 4 μm were prepared from buffered formalin-fixed, paraffin-embedded tissue blocks. Immunohistochemistry (IHC) for fibroblast activating protein (FAP) was performed on the tissue sections with a primary monoclonal antibody (clone SP325, abcam, dilution 1:100) on an automated staining system (Ventana Discovery XT, Munich, Germany) according to standard protocol using the OptiView DAB detection kit. Briefly, pretreatment for antigen retrieval was performed by heating in citrate buffer (Ultra Cell Conditioning Solution II, Ventana Medical Systems, Basel, CH) at pH 6, 90°C for 48 min followed by antibody incubation for 60 min at 36°C. Assessment of immunohistochemical staining of the TMAs were evaluated by two different pathologists who were both blinded regarding the clinical data and outcome of the patients. The number of FAP-positive staining was counted in the whole tissue cores and the percentage of positive cells was calculated. As suggested by Henry et al. [24] immunoreactivity was evaluated semi-quantitatively, considering greater than 1% and less than 10% FAP-positivity in stromal cells as low expression and samples with at least 10% FAP-positive cells as high expression. Additionally, for each case the immunoreactive score (IRS) was determined, which is a multiplication score combining staining intensity and percentage of positive cells. The FAP staining intensity was visually scored and stratified as follows: 0 (negative), 1 (weak), 2 (moderate) and 3 (strong). Points for the percentage of positive cells were assigned as follows: 0: none, 1: 1%–10%, 2: 11%–50%, 3: 51%–80%, and 4: 81%–100%. These two values were then multiplied together to determine the IRS (ranging from 0 to a maximum of 12). IRS value 0 was considered FAP-negative whereas IRS values 1 to 4 were used to define the low expression group and values 5 to 12 the high expression group of FAP.

To determine the MSI status, IHC analysis was conducted according to established protocols, as previously described using standard techniques [25]. The specimens were categorized as MMR-proficient or MMR-deficient based on the presence or absence of nuclear stains of MMR proteins (MSH2, MSH6, MLH1 and PMS2). In certain cases, additional polymerase chain reaction (PCR) was performed using a commercially available five-marker MSI testing kit (Promega, United States). Evaluation of p53, β-catenin (BCAT) and Ki67 was depicted with the percentage of stained cells. Evaluation of CD3 was performed with a four-tier score: 0 described no infiltration, 1 described low infiltration, 2 described intermediate infiltration and 3 described high infiltration. Additionally, nuclear β-catenin aberrance was evaluated. An overview of all used clones, dilutions and pretreatments is given in Supplementary Table S1.

### Statistical analysis

Statistical analysis was performed using the R statistical programming environment V 4.0.2. Before starting exploratory data analysis, normal distribution of each data set was tested by Shapiro-Wilks-test taking ordinal as well as metric variables into account. Either two-sided Students’s t-test (parametric) or Wilcoxon rank-sum test (non-parametric) was used for dichotomous variables. To compare factors with more than two groups or variables with ordinal character, either ANOVA (parametric) or Kruskal-Wallis test (non-parametric) was performed.

Analysis of double dichotomous contingency tables was carried out using Fisher’s exact test. Pearson’s Chi-squared test was used to assess dependency of ranked parameters with more than two groups.

Due to the use of multiple statistical tests the *p*-values were adjusted by using the false discovery rate (FDR). A value of *p* ≤ 0.05 after adjustment was considered statistically significant.

## Results

To evaluate the overall FAP expression in CRC, a cohort of resected adenocarcinomas, low- and high-grade adenomas, along with tissue samples of inflammation sites as well as non-inflammatory, non-neoplastic specimens of colonic mucosa were analyzed by immunohistochemistry. The quality of study material was heterogeneous, resulting in a variable subset of the cohort (*n* = 137) being evaluable for IHC analysis. Evaluable samples were available from 67 adenocarcinomas, 24 adenomas, 18 colonic inflammation specimens and 28 non-neoplastic, non-inflammatory tissue samples.

**FIGURE 1:**
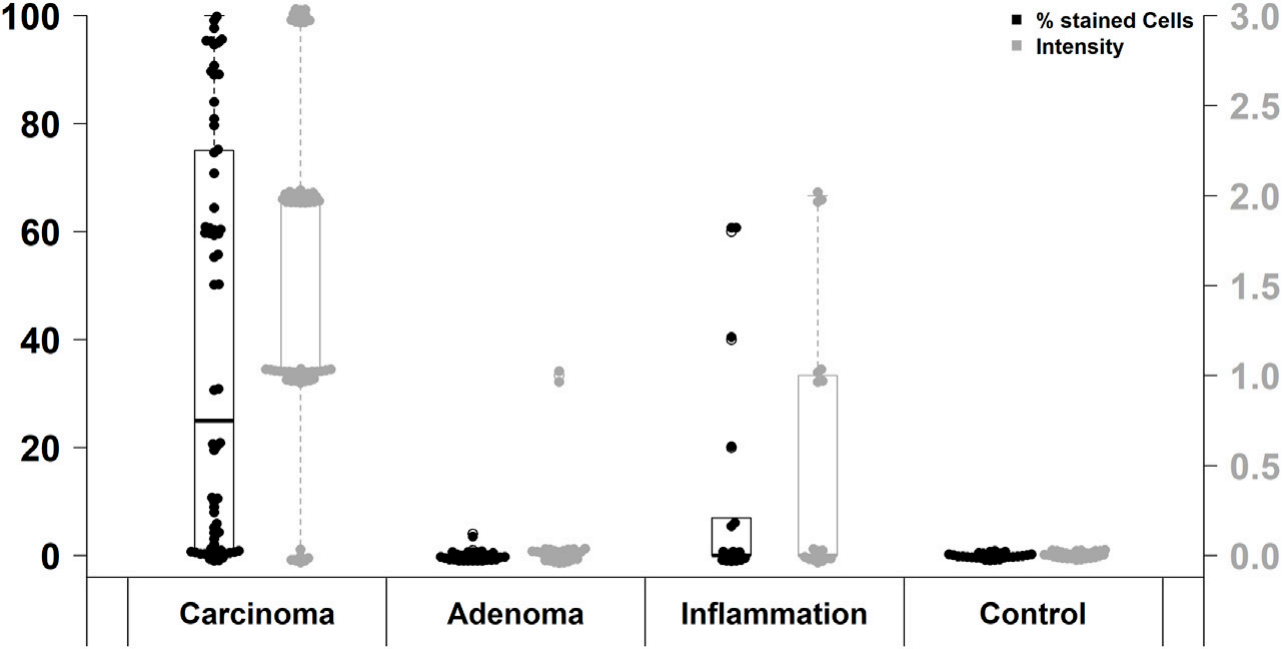
Immunohistochemical FAP staining levels in different investigated groups.

FAP expression was observed in 53 of 67 (79%) CRC cases, while positive FAP expression was also detected in 6 of 18 (33%) inflammatory tissue specimens. However, FAP was only present in 1 of 24 (4%) adenomas and absent in non-neoplastic, non-inflammatory tissue (0/28). Thus, FAP expression was significantly higher in CRC than in the other investigated groups (*p* < 0.0001; Pearson’s Chi-squared Test). In more detail, significant differences in FAP expression were observed when considering negative, low positive, and high positive cases. These differences were evident between CRC and adenomas (*p* < 0.0001; χ^2^ = 41.8), CRC and inflammation specimens (*p* < 0.0001; χ^2^ = 28.9), as well as CRC and non-neoplastic, non-inflammatory tissue (*p* < 0.0001; χ^2^ = 35.8). Moreover, there was a significantly higher level of FAP expression in inflammatory sites compared to adenomas (*p* = 0.02905; χ^2^ = 7.1). Results are presented in Table 1 and Figure 1.

**TABLE 1:** FAP expression in different investigated groups.

Specimen	Cases (n)	FAP Expression semi-quantitatively total (%)		Positive FAP Expression (semi-quantitatively)		FAP Expression IRS total (%)		Positive FAP Expression (IRS)	
		Positive	Negative	High	Low	Positive	Negative	High	Low
Carcinoma	67	53 (79.1)	14 (20.9)	41	12	42 (62.7)	25 (37.3)	28	14
Adenoma	24	1 (4.2)	23 (95.8)	0	1	0 (0)	24 (100)	0	0
Inflammation	18	6 (33.3)	12 (66.6)	4	2	6 (33.3)	12 (66.6)	1	5
Non-neoplastic, non-inflammatory tissue	28	0 (0)	28 (100)	0	0	0 (0)	28 (100)	0	0

Immunoreactivity was evaluated semi-quantitatively, considering greater than 1% and less than 10 % FAP-positivity in stromal cells as low expression and samples with at least 10% FAP-positive cells as high expression. The immunoreactive score (IRS) was determined by multiplying the ratio of positive-stained fibroblasts (0: none, 1: 1%–10%, 2: 11%–50%, 3: 51%–80%, and 4: 81%–100%) and the staining intensity (0: no staining, 1: weak, 2: moderate, and 3: strong). The total IRS ranges from 0 to 12. IRS value 0 was considered FAP-negative whereas IRS values 1–4 were used to define the low expression group and values 5–12 the high expression group of FAP.

**FIGURE 2:**
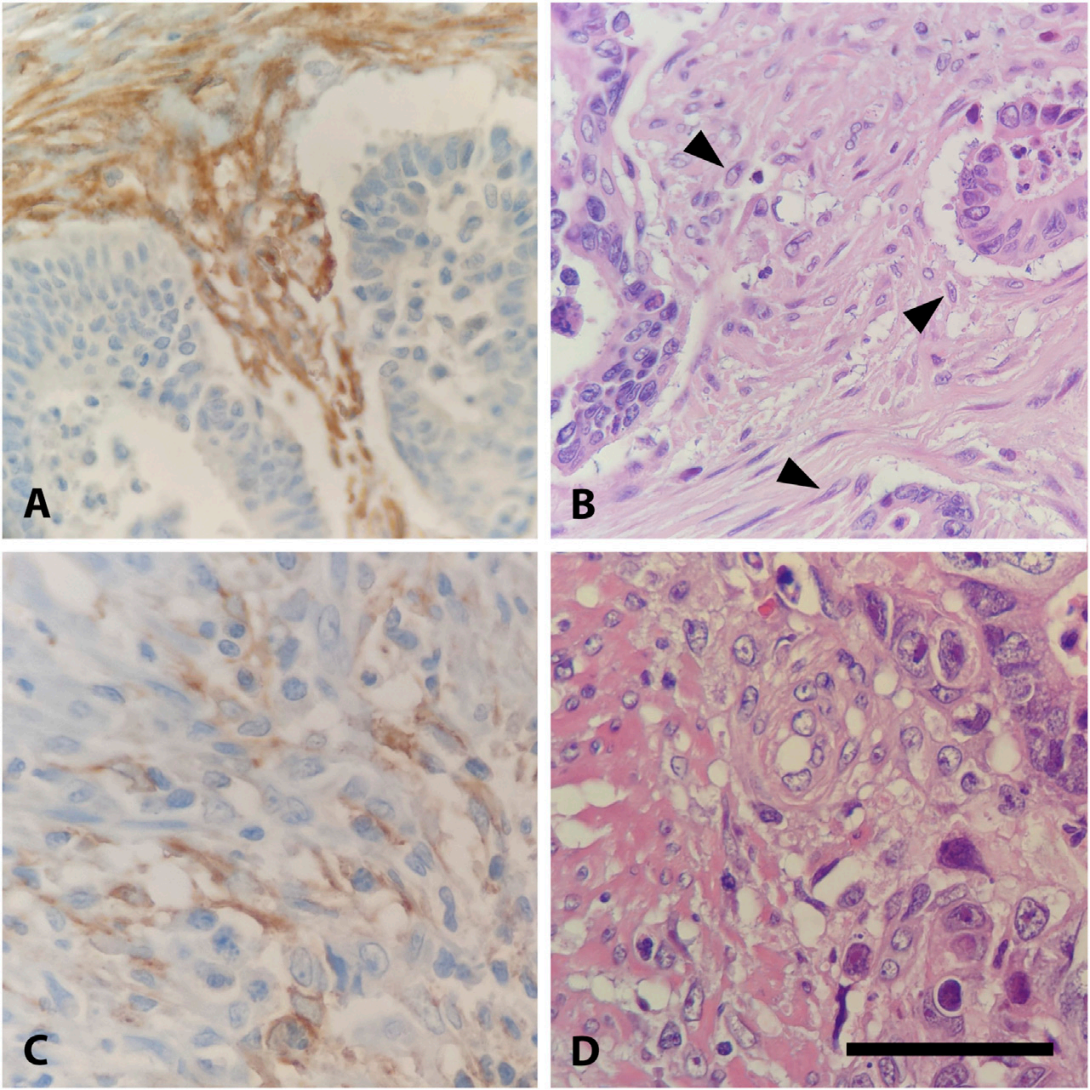
Expression of FAP in CRC. **(A)** High FAP expression in stromal fibroblasts of an intermediate grade adenocarcinoma, ×400. **(B)** Hematoxylin and Eosin (H&E) stain of **(A)** with activated fibroblasts, identified by typical morphological features: increased cell size, altered nuclear morphology, and enhanced cytoplasmic basophilia (highlighted with arrowhead), ×400. **(C)** Low stromal expression of FAP in an intermediate grade adenocarcinoma, ×400. **(D)** Hematoxylin and Eosin (H&E) stain of C, ×400. Scalebar is 50 μm.

41 of 53 (77%) FAP-positive CRC showed high expression levels and 12 (23%) showed low expression in semi-quantitative evaluation. Regarding the immunoreactive score (IRS), only 42 of 67 (63%) CRC specimens were classified as FAP-positive, of which 28 (67%) had a high expression and 14 (33%) a low expression of FAP. The difference between high and low FAP expression was neither in semi-quantitative assessment nor in IRS evaluation statistically significant (Table 1). In the evaluation of the percentual expression of FAP in the two different tumor cores on the TMA, a deviation of more than 5% was detectable in 29 CRC cases (43%). Figure 2 shows representative examples of high and low immunohistochemical expression of FAP in CRC.

Within the CRC cohort, expression of FAP did not correlate with tumor stage or grading (Supplementary Table S2). FAP positive specimens more frequently showed lymph node metastases, however, the difference was not statistically significant (21/45, 47% and 2/11, 18%, respectively, *p* = 0.074; Wilcoxon rank-sum test). Equally, no significant difference in the occurrence of distant metastases could be shown (11/31, 35% and 1/2, 50%, respectively, *p* = 0.2; Wilcoxon rank-sum test).

**FIGURE 3:**
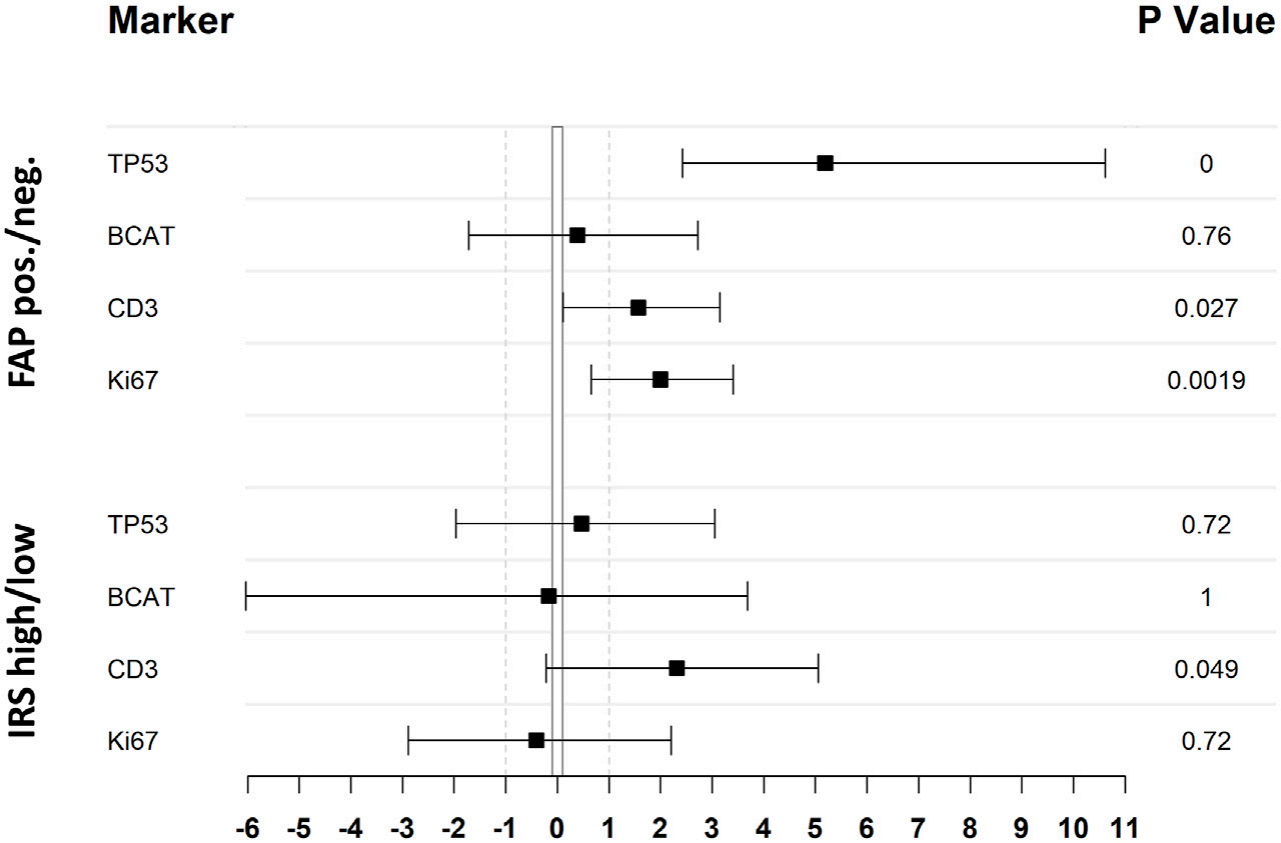
Statistical analysis of the association between expression of investigated IHC markers and FAP expression illustrated as odds-ratio forest plot. Black squares indicate the odds ratio (log2 transformed) whereas the black lines refer to the 95% CI. Upper four lines result from a comparison of FAP positive and negative cases, lower four lines differentiate between cases with low or high IRS score as stated in the material and methods section. TP53 and BCAT expression has been classified as aberrant and not-aberrant, whereas CD3 and Ki67 has been grouped in high and low expression (cut-off CD3: > 1; cut-off Ki67: > mean [47.1]) to achieve double-dichotomous contingency tables. All *p*-values where calculated by Fisher’s exact test.

**FIGURE 4:**
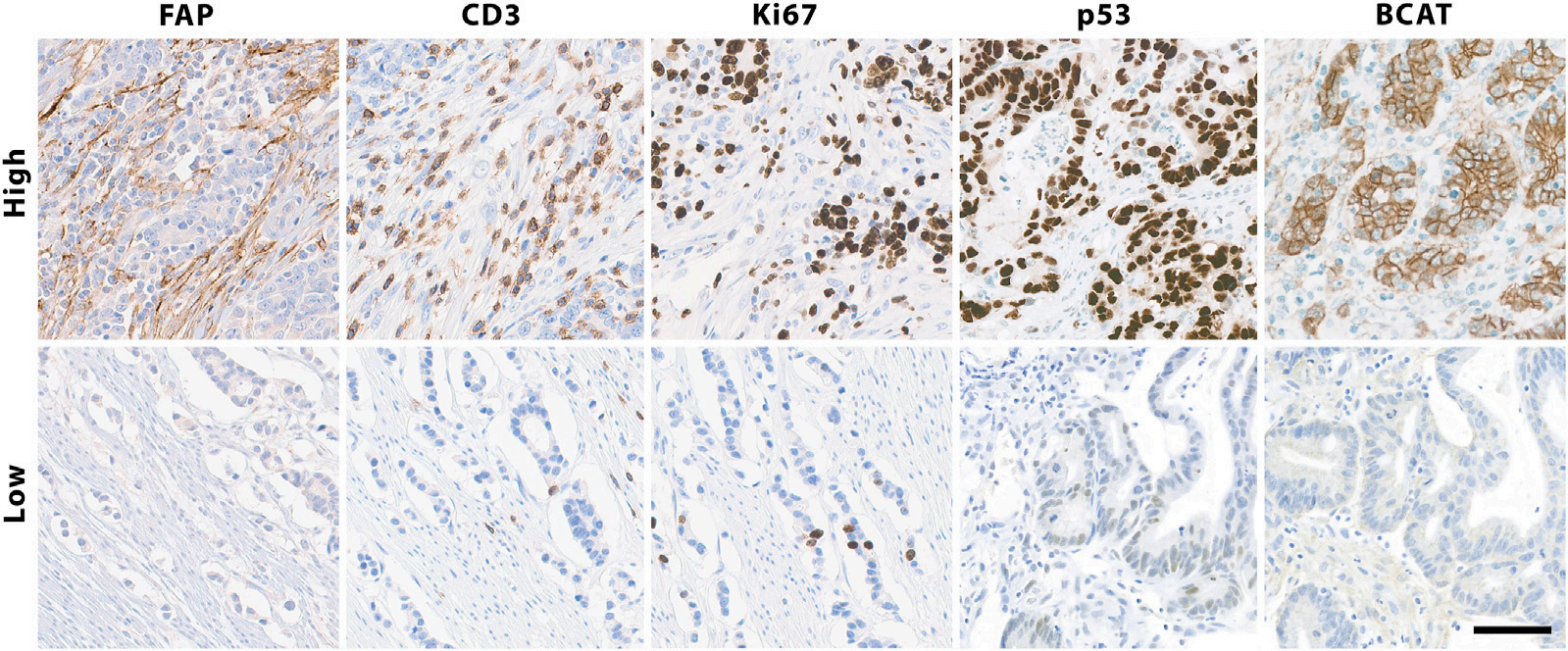
Immunohistochemical staining of colorectal cancer tissue for FAP, analyzed as either high or low expression, considering greater than 1% and less than 10% FAP-positivity in stromal cells as low expression and samples with at least 10% FAP-positive cells as high expression, as well as high and low levels of Ki-67, CD3, p53 and β-Catenin, exemplified in top boxes, ×200. Scalebar is 50 μm.

There was no correlation observed between FAP expression and the patients’ age, gender, or the primary site of the tumor (Supplementary Table S2). However, CRC tumor cores with positive expression of FAP also showed higher expression of other immunohistochemical parameters: There was a significant correlation between percentual FAP positivity and the expression of tumor protein p53 (*p* < 0.0001, Wilcoxon rank-sum test with continuous TP53 values; *p* < 0.0001, Fisher’s exact test with grouped TP53 values (aberrant/non-aberrant)). Similarly, a significant correlation was found between FAP positivity and the expression of Ki-67, a nuclear protein that is associated with cellular proliferation (*p* < 0.0001, Wilcoxon rank-sum test with continuous Ki67 values; *p* = 0.0019, Fisher’s exact test with grouped Ki67 values (high/low)). Additionally, FAP positive tumors more frequently displayed elevated numbers of tumor-infiltrating CD3-positive T-lymphocytes [*p* = 0.014; Wilcoxon rank-sum test with ordinal CD3 values; *p* = 0.027, Fisher’s exact test with grouped CD3 values (high/low)]. This trend was also observed when comparing cases with high and low IRS (*p* = 0.049, Fisher’s exact test). Regarding the immunohistochemical expression of β-Catenin, higher levels were observed in FAP-positive tumors on a continuous scale of expression intensity (*p* = 0.02; Pearson’s LM/Product Moment Correlation). However, this association could not be confirmed when comparing samples with aberrant and normal expression patterns of BCAT, where the localization of BCAT (nuclear vs. cytoplasmic) needed to be considered (*p* = 0.76, Fisher’s exact test). An overview of statistical results of the immunohistochemical markers are presented in Figure 3. Regarding the association between FAP expression and the microsatellite instability (MSI) status of the tumors, no significant correlation could be shown (Supplementary Table S2). Out of the 52 FAP positive tumors with available immunohistochemical MSI status, 8 (15%) exhibited deficient MMR protein expression. Conversely, among the 11 tumors with negative FAP expression, 2 (18%) were also diagnosed as MMR-deficient (*p* = 0.6732, Fisher’s Exact Test). PCR results for MSI status were only available for a subset of colorectal carcinomas (*n* = 14). Among this subset, 8 out of 13 FAP positive carcinomas displayed high microsatellite instability (MSI-high). Nonetheless, it is worth noting that also one FAP negative tumor with available PCR results was classified as MSI-high, so no significant difference could be observed. Representative examples of immunohistochemistry corresponding to FAP expression are shown in Figure 4.

## Discussion

In the context of developing new therapeutic approaches for combating CRC, the focus of attention lies not merely on the neoplastic cells anymore but also on the TME and the crosstalk between tumoral and stromal cells. FAP expressing cancer-associated fibroblasts (CAF) are the predominant component in the stroma of CRC and have been recognized as co-responsible for tumor growth and metastasis.

The objective of this study was to investigate FAP expression of CAF in the tumor microenvironment of CRC and to evaluate FAP as a candidate for targeted imaging and therapy.

Consistent with previous reports, expression of FAP was detectable in the majority of CRC but nearly absent in precursor lesions (i.e., adenomas) and non-neoplastic, non-inflammatory tissue [8, 26, 27]. This finding supports the role of FAP in tumor growth and invasion. Moreover, in this study, a positive correlation between FAP expression and several immunohistochemical markers that are involved in carcinogenesis could be shown.

Tumors with expression of FAP significantly more often displayed high expression of Ki-67, a nuclear protein that is associated with cellular proliferation. Similarly, an association with immunohistochemical expression of p53 was detected. This has previously also been reported for non-small cell adenocarcinomas of the lung [28]. p53 is a tumor suppressor protein that regulates cell growth by promoting cell apoptosis and DNA repair. When becoming mutated p53 loses its function which results in accumulation of the protein in the cell promoting an abnormal cell proliferation and tumor progression [29]. This emphasizes the involvement of FAP in crucial processes of tumor development. Another parameter associated with positive FAP expression in this study was β-Catenin. It is involved in regulation and coordination of cell–cell adhesion and gene transcription and acts as an intracellular signal transducer in the Wnt-signaling pathway as a subunit of the cadherin protein complex [30]. Moreover, Wnt-signaling has been shown to be a key feature in the crosstalk between activated myofibroblasts and cancer cells. Overexpression of β-catenin is associated with many cancers, including CRC [30]. Studies have demonstrated that FAP expression in the membrane of CAF induced this β-Catenin related pathway in CRC [3, 18]. Previous investigations also displayed that combined expression of FAP and β-Catenin was independently associated with the occurrence of metastasis [18].

In addition to the findings above, in this study FAP positivity also correlated with high expression of CD3 on T-lymphocytes in the tumor stroma of CRC. Several studies have indicated that FAP expression is associated with a shift in immune cell populations within the tumor, thereby promoting a pro-tumorigenic environment [31, 32]. Here, only one immune cell parameter has been evaluated, so no final conclusions can be drawn. But the results point out the value of further investigating the link between CAF and immunoreactivity in CRC. The described association between FAP and the expression of the immunohistochemical parameters mentioned above displays the interaction between different components of the TME. Interestingly, while a positive correlation was observed between FAP expression and other immunohistochemical parameters, no significant association between FAP expression and MSI status of the tumors was found in this investigation. It should be noted that the number of CRC cases with microsatellite instability in this study was relatively small (9 MSI-H tumors detected in PCR and 10 MMR-deficient tumors detected in IHC, respectively), which may have influenced the lack of correlation. On the other hand, CRC with MSI are generally less aggressive than MSS tumors and exhibit a lower incidence of lymph node metastases or distant spread [26], which could potentially explain the lower expression of FAP in these tumors. To clarify this coherence, further investigation is needed.

In line with other studies that evaluated FAP expression by IHC [8, 27], FAP was not correlated to age and gender of the patients nor to the primary site of the tumor. In this study, no correlation between FAP expression and the tumor stage and grading could be found and no significant correlation was observed between FAP expression and the occurrence of lymph node or distant metastases. However, it should be noted that the number of metastatic events, particularly in the group of FAP negative tumors, was limited, making it difficult to draw definitive conclusions.

Despite the fact that FAP was widely present in carcinomas but nearly absent even in high-grade adenomas and not detectable in non-neoplastic, non-inflammatory tissue, it could serve as a diagnostic marker in routine diagnostics of early CRC and be helpful to discriminate invasive carcinoma from high-grade dysplasia in small biopsies or adenomatous polyps. In the recent years, there is an increasing use of nanomedicine in cancer including radionuclide-based approaches for targeted therapy. Recently, the FDA approved 68Ga-PSMA as first PSMA-targeted PET drug in potentially curable patients with metastatic prostate cancer [33]. In addition, endo-radioligand therapy with Lu177-PSMA has been proven powerful in therapy of advanced prostate cancer [34]. Beside PSMA, there are multiple other targets discussed for their potency in PET/CT diagnostics or radioligand therapy. PET imaging using a 64Cu- or 89Zr-labeled monoclonal antibody against mesothelin as well as therapeutical usage of antibody-drug conjugate treatment has shown promising results in pancreatic and/or ovarian cancer [35, 36]. 68Ga-Pentixafor as radio ligand for CXCR4 has been proven an alternative to 18F-FDG PET, showing clearly higher detection rates and better tumor-to-background contrast [37]. It also has to be discussed as potential candidate for directed endo-radiotherapy [38].

The fibroblast activating protein (FAP) is used in 68Ga-FAPI PET/CT [39, 40] as well as 64Cu- and 225Ac-labeled FAPI combined in a “theranostics” approach [41]. Surprisingly, Kratochwil et al. [42] demonstrated colorectal and pancreatic carcinomas only with intermediate 68Ga-FAPI uptake, although they showed highest amounts of DSR histologically. However, they reported significant lower hepatic background compared to 18F-FDG and therefore an advantage for the detection of liver metastases. A case report using [68Ga]Ga-DOTA.SA.FAPI PET/CT-guided [177Lu]Lu-DOTA.SA.FAPI radionuclide therapy in a patient with metastatic (lung, liver, bones and brain) end-stage breast cancer showed intense radiotracer accumulation in all lesions in concordance to PET/CT [43]. As post treatments, symptoms and laboratory parameters decreased with no treatment-related adverse events, the authors concluded this as new opportunity in breast cancer therapy for patients refractory to conventional treatment options. Additionally, in head and neck cancer, evidence of diagnostic and therapeutic potential of FAPI ligands could be proven, although no histopathological DSR correlate or FAP IHC has been evaluated in upfront diagnostics [44]. In recent studies, FAPI tracers are already successfully developed for SPECT imaging and combined therapy in a theranostics setting, since SPECT is a lower-cost and broader available technology compared to PET [45]. Of note, “next-generation” of FAPI tracers are on their way, showing improved tumor retention and pharmacokinetics [46].

Given the fact that FAP proved to be a reliable marker of activated CAF, and that high expression of FAP almost exclusively occurs in invasive carcinomas but not in adenomas or non-neoplastic, non-inflammatory tissue, FAP has the potential to emerge as a powerful target for new diagnostic and therapeutic concepts in CRC. However, because the amount of available data is limited, further research in this field is necessary. When considering FAP as a promising tool in new cancer diagnostics and therapeutics, one should also take into account the immunohistochemical scoring system that is used to quantify the FAP expression. Up until now, there is no standard quantification score for an objective evaluation of CAF activation markers such as FAP, so studies lack comparability. In the present study, there were different results depending on the scoring method that was used. In particular, the positive rate (including high and low expression) of FAP in CRC was 62.6% using the IRS system to quantify the immunohistochemical expression which was different from the 79.1% positivity using the semi-quantitative grading system suggested by Henry et al. [24] (Table 1). Regarding the FAP positivity in inflammation sites there was no difference between the scoring systems. Although examination of FAP positivity on TMAs has proved to be a fast and reliable procedure [8, 27], for further research and for establishing a standardized scoring system whole slide evaluation of FAP should be preferred.

## Conclusion

In the crosstalk between tumor cells and TME, FAP expressing CAF play a key role in carcinogenesis and metastatic spread. In this study, FAP proved to be a suitable marker for activated CAF in CRC. With regard to the urgent need for new strategies in cancer treatment, FAP may serve as a possible target for new diagnostics and targeted therapy of CRC in the near future.

## Data Availability

The raw data supporting the conclusion of this article will be made available by the authors, without undue reservation.

## Abbreviations

CRC, colorectal cancer; CAF, cancer-associated fibroblasts; ECM, extracellular matrix; FAP, fibroblast activation protein-α; IHC, immunohistochemistry; IRS, immunoreactivity score; MSI, microsatellite instability; MSI-H, High microsatellite instability; MSS, microsatellite stability; MMR, mismatch repair; PDGF-R, platelet-derived growth factor receptor; TMA, tissue microarray; TME, tumor microenvironment.
