# Supplementary material for: Fibroblast activation protein-α expression in fibroblasts is common in the tumor microenvironment of colorectal cancer and may serve as a therapeutic target
Source: Pathol Oncol Res. 2023 Aug 8;29:1611163. doi: 10.3389/pore.2023.1611163 (PMC10442481; doi:10.3389/pore.2023.1611163)
Supplement: Supplementary file 1 [file Table1.docx]

| **Target** | **Clonality** | **Dilution** | **Antigen retrieval** | **Incubation** | **Manufacturer** | **Clone** |
| --- | --- | --- | --- | --- | --- | --- |
| p53 | Mouse  Monoclonal | 1:1000 | CC1 90°C  56 min | 24 min at 37 °C | Zytomed Systems | BP53-12 |
| β-catenin | Rabbit Polyclonal  (AB_2864620) | 1:1000 | CC1 90°C  16 min | 24 min at 37 °C | Zytomed Systems | - |
| Ki67 | Rabbit Monoclonal | Ready to use | CC1 90°C  32 min | 16 min at 37 °C | Roche | 30-9 |
| CD3 | Rabbit Monoclonal | 1:200 | CC1 90°C  40 min | 24 min at 37 °C | DCS | SP7 |
| FAP | Rabbit Monoclonal | 1:100 | CC1 90°C  48min. | 36°C 60min. | abcam | SP325 |
| MSH2 | Mouse  Monoclonal | Ready to use | CC1 100°C 40min. | 36°C 12min. | Roche | G219-1129 |
| MSH6 | Mouse  Monoclonal | 1:100 | CC1 90°C 32min. | 36°C 24min. | DCS Innovative Diagnostic-Systems | 44 |
| MLH1 | Mouse  Monoclonal | 1:75 | CC1 90°C 72min. | 36°C 60min. + Amplification 4min. | Zytomed Systems | G168-15 |
| PMS2 | Rabbit Monoclonal | 1:25 | CC1 90°C 72min. | 36°C 100min. | DAKO | EP51 |

**Supplementary table 1:** Overview of all used clones, dilutions and pretreatments.

CC1: Ventana's proprietary antigen retrieval solution.
