# Supplementary material for: Fibroblast activation protein-α expression in fibroblasts is common in the tumor microenvironment of colorectal cancer and may serve as a therapeutic target
Source: Pathol Oncol Res. 2023 Aug 8;29:1611163. doi: 10.3389/pore.2023.1611163 (PMC10442481; doi:10.3389/pore.2023.1611163)
Supplement: Supplementary file 2 [file Table2.docx]

**Supplementary table 2:** FAP expression within the CRC cohort (n=67)

| **Category** | **Cases (n)** | **FAP Expression** | | | **p-value** |
| --- | --- | --- | --- | --- | --- |
|  |  | **high** | **low** | **negative** |  |
| **Age**  < 65 years  > 65 years | 29  38 | 18  23 | 6  6 | 5  9 | 0.063^1^ |
| **Gender**  Female  Male | 33  34 | 18  23 | 7  5 | 8  6 | 0.078^2^ |
| L**ocalization**  Caecum/Ascendens  Transversum  Descendens/Sigma  Rectum  n/a | 28  7  21  8  3 | 16  2  15  6  2 | 7  0  4  1  0 | 5  5  2  1  1 | 0.6^3^ |
| **Grading**  G1  G2  G3  n/a | 1  46  14  6 | 1  32  7  1 | 0  5  4  3 | 0  9  3  2 | 0.88^1^ |
| **T-Stage**  T1  T2  T3  T4  n/a | 6  9  36  7  9 | 2  4  26  3  6 | 1  3  5  3  0 | 3  2  5  1  3 | 0.84^1^ |
| **N-Stage**  N0  N1  N2  n/a | 33  14  9  11 | 20  10  4  7 | 4  2  5  1 | 9  2  0  3 | 0.074^1^ |
| **M-Stage**  M0  M1  n/a | 21  12  34 | 18  7  16 | 2  4  6 | 1  1  12 | 0.2^1^ |
| **MMR protein IHC**  MMR-deficient  MMR-proficient  n/a | 10  53  4 | 6  34  1 | 2  10  0 | 2  9  3 | 0.6732^2^ |
| **MSI PCR**  MSI-high  MSI-low  MSS (stable)  n/a | 9  0  5  53 | 6  0  3  32 | 2  0  2  8 | 1  0  0  13 | 1.0^2^ |

FAP expression in greater than 1% and less than 10 % of stromal cells was considered low expression and samples with at least 10% FAP-positive cells as high expression.

^1^ Wilcoxon rank-sum test, ^2^ Fisher’s Exact Test, ^3^ Pearson’s Chi-squared Test
